# Focus on HPV Infection and the Molecular Mechanisms of Oral Carcinogenesis

**DOI:** 10.3390/v13040559

**Published:** 2021-03-26

**Authors:** Luigi Santacroce, Michele Di Cosola, Lucrezia Bottalico, Skender Topi, Ioannis Alexandros Charitos, Andrea Ballini, Francesco Inchingolo, Angela Pia Cazzolla, Gianna Dipalma

**Affiliations:** 1 Interdisciplinary Department of Medicine, Microbiology and Virology Unit, School of Medicine, University of Bari “A. Moro”, 70124 Bari, Italy; luigi.santacroce@uniba.it; 2 Interdepartmental Research Center for Pre-Latin, Latin and Oriental Rights and Culture Studies (CEDICLO), University of Bari “A. Moro”, 70121 Bari, Italy; bottalico.lu@gmail.com (L.B.); skender.topi@uniel.edu.al (S.T.); 3 Department of Clinical Disciplines, School of Technical Medical Sciences, “A. Xhuvani” University of Elbasan, 3001 Elbasan, Albania; 4 Department of Clinical and Experimental Medicine, Università degli Studi di Foggia, 71122 Foggia, Italy; michele.dicosola@unifg.it (M.D.C.); elicio@inwind.it (A.P.C.); 5 Department of Biosciences, Biotechnologies and Biopharmaceutics, Campus Universitario “G. Quagliarello”, University of Bari “A. Moro”, 70125 Bari, Italy; 6 Department of Precision Medicine, University of Campania “Luigi Vanvitelli”, Vico L. De Crecchio 7, 80138 Naples, Italy; 7 Interdisciplinary Department of Medicine, University of Bari “A. Moro”, 70124 Bari, Italy; francesco.inchingolo@uniba.it (F.I.); giannadipalma@tiscali.it (G.D.)

**Keywords:** oral microbiota, oral infections, HPV, oral carcinogenesis, oral cancer, maxillary-facial surgery

## Abstract

This study is focused on the epidemiological characteristics and biomolecular mechanisms that lead to the development of precancerous and cancerous conditions of oral lesions related to Human Papilloma Virus (HPV) infections. Current evidence from the literature demonstrates the role of HPV in potentially malignant oral disorders. Therefore, the underlying biomolecular processes can give arise, or contribute to, benign lesions as well as to oral carcinogenesis.

## 1. Introduction

Oral diseases, their symptoms and therapy have already been observed and described by the ancient doctors with a scientific approach by Hippocrates of Kos (Ἱπποκράτης ὁ Κῷος 460–370 BC), Galen of Pergamum (Γαληνός ὁ Περγαμηνός, 130–200 BC), Oribasius of Pergamum (Oρειβάσιος ὁ Περγαμηνός, 325–403 AD), and others [1]. Oral squamous cell carcinoma (SCC) is the sixth most common cancer worldwide according to the latest WHO report. SCC of the lips/mouth is one of the three most common cancers in some countries of Asia and the Pacific. It can also develop in the larynx and pharynx, and a congenital form is also present [2,3]. Mouth cancer can be in several areas including the lips, inside the cheeks, the floor or palate of the gums, and the tongue. Symptoms of SCC of the mouth can include a persistent ulcer and/or lump, and both can be painful [3]. Instead, salivary gland malignant tumor is rare, and thus, is not considered among the tenth major cancers [2,3]. The incidence of oral cancer (age-adjusted) in the world is 4 cases per 100,000 people, with wide variation across the globe which depends on gender, age, countries, races, and socio-economic condition (20 out of 100,000 cases) [3]. In 2018, the number of new cases for both sexes and age groups was 354,864, with 177,384 number of deaths in the same year [2,3]. In some Asian-Pacific countries, such as India and Pakistan, the incidence of oral cancer in 2018 ranked among the three top cancers. The most involved areas of the world have been South-Central and Eastern Asia (64.2%), followed by Central and Eastern Europe (17.4%), North America (7.6%); particularly, oral cancer is the most common malignancy in India among men and the fifth most common in females. On the other hand, Latin America and the Caribbean, Africa, and Oceania present a low incidence. Mortality for oral cancer follows similar percentage in those countries (73.3% in Asia, 13.6% in Europe). Its high mortality rate is dependent, above all, on the stage of the disease at diagnosis, which is often already advanced. In 2020, lip and oral cavity cancer was the sixteenth cancer and the fifteenth cause of death worldwide [1,3].

## 2. The Established Risk Factors for Oral Cancer

**Figure 1:**
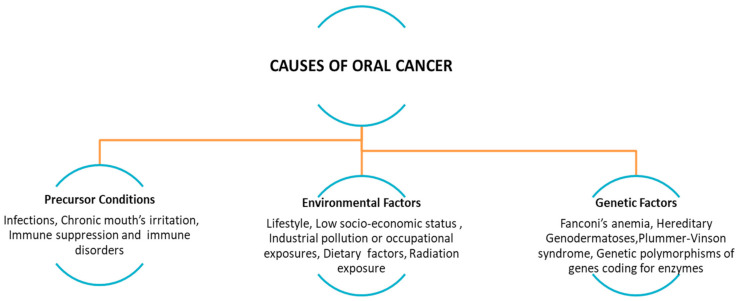
Precursor conditions: infections, chronic mouth’s irritation, immune suppression and immune disorders (i.e., transplanted patients, due to the chronic inflammatory state associated with graft-versus-host disease (GVHD), lifestyle and environmental factors (e.g., alcohol abuse, tobacco smoking/chewing, betel quid or guṭkha chewing, marijuana, poor oral hygiene), low socio-economic status (poor or no access to oral health care facilities), dietary factors, radiation exposure, and genetic polymorphisms. (Adapted from [1]).

Globally, oral cancer’s risk increases with age and it is more frequent in male than female, as well as its global mortality rate. Oral SCC is among the most common head and neck cancer (HNSCC) [2,4,5]. Most of the different frequencies of oral cancer between the developing world and the Western world are attributable to several causes: different habits of the population, education about prevention, oral hygiene, and the quality of health services affected by poverty, illiteracy, lack of access to health-care facilities, and poor quality of treatment. An additional difference between countries is the vaccination program as well as the type of vaccine (i.e., quadrivalent and nonavalent vaccine) [2,5,6]. Tobacco (including the use of cigarettes, cigars, pipes, beedi, chewing tobacco, and snuff use), alcohol (in relation to dose-response, frequency and duration of its use), and betel quid (with or not tobacco use) are well-established risk factors for oral cancer that have been known for decades. While a few studies have focused on e-cigarettes smoking and risk of oral cavity cancer. The other factors that can locally and systemically favor the onset of oral cancer may be malocclusions, fillings or incongruous prostheses, bad oral hygiene, food deficiencies (such as disvitaminosis A and C), irritating substances and foods, trauma, hereditary diseases (dyskeratosis congenita), viral, bacterial, fungal (chronic candidiasis), states of immunosuppression (congenital or acquired), and others. Bacteria, in particular *Helicobacter pylori*, viruses (such as those belonging to the Papillomaviridae and Polyomaviridae family) and fungi (i.e., *Candida albicans*) are associated with some types of cancer (Figure 1) [1,6,7,8,9,10].

## 3. The Human Papillomaviruses (HPVs)

**Figure 2:**
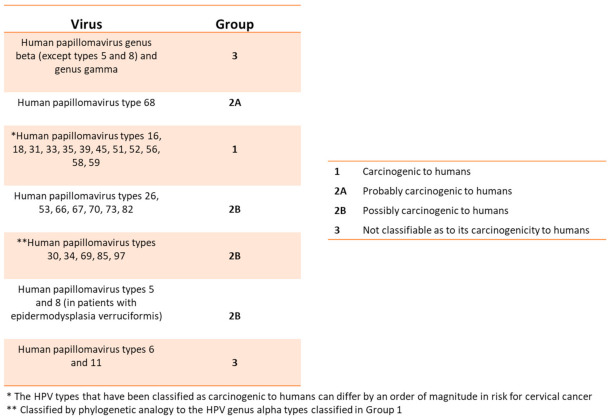
This table relates the HPVs with the different degrees of risk for developing precancerous lesions or cancer (From WHO/IARC Agents Classified by the IARC Monographs, IARC Monographs on the identification of carcinogenic hazards to humans, until 2019).

HPVs are members of the Papillomaviridae (PV) family. To date, over 450 distinct types of human (HPV) belong to this virus family. The HPV viral body consists of a circular, double-stranded, and supercoiled DNA whose length is 7900 bp and a weight limit of approximately 5.2 × 10^6^ Dalton [11,12,13,14]. It is surrounded by a hexahedral symmetry protein capsid composed by 72 pentameric capsomers and contains only two structural proteins, L1 and L2 HPV, is considered an oncogenic virus accounting for several clinical manifestations including cancer. In fact, other infections (such as the hepatitis C virus or the *Helicobacter* *pylori*) can cause malignant tissue degeneration over time [11,12,13]. HPVs affect the skin and mucous membranes, causing benign hyperplastic lesions and precancerous and cancerous lesions. The International Committee on Taxonomy of Viruses (ICTV) officially recognizes 2 subfamilies, 53 genera, and 133 species of the Papillomaviridae. The HPV’s genotypes are divided according to the oncogenic risk from the International Agency for Research on Cancer (IARC)/WHO (Figure 2) and are associated with precancerous lesions or cancer [11,14,15].

Most of these viruses are low risk and are associated with benign lesions such as papillomas, warts, wedges, and Heck disease (focal epithelial hyperplasia). Malignant lesions by carcinogenic HPV are anal, genital (penile, vulval, vaginal, cervical), and some types of oral. HPV tumor risk strains are mostly associated with epithelial dysplasia and squamous cell carcinomas and metastasis. The genotypes 1, 2, 3, 4, 6, 7, 10, 11, 13 are found in various type lesions of the oral cavity [3,5,14]. An additional potential risk factor for HPV-driven oral carcinogenesis might be represented by the infection of the upper respiratory tract by oncogenic HPVs. Indeed, several works reported the presence of HPV16 DNA/mRNA in nasal/Killian polyps nearby the maxillary sinus region. These studies suggest that, HPV16 can potentially drive malignant transformation of the oral region following nasal polyp infection [16,17]. The HPV molecular test aims to search for viral DNA sequences in tissues. Cells analyzed are the same such as those observed with the Papanicolaou test (Pap test). Furthermore, the association between HPV and oral squamous cell carcinoma, on the other hand, is sometimes controversial by some studies in literature, whih have shown that this association would be rare. However, the different and conflicting results thus obtained depends on two reasons. The first depends on the case history of some studies that do not distinguish between cancer of the oropharynx and the anterior oral cavity (these two tumors have quite different biological aspects) [18,19,20,21,22]. The second, on the other hand, depends on the different sampling techniques (saliva, biopsies, and brushing) and those for the detection of the various viral gene sequences such as polymerase chain reaction or PCR, dot-blot hybridization, and Southern blotting [12,23,24,25,26,27]. As the novel HPV DNA detection method is the highly sensitive droplet sensitive PCR (ddPCR). Previous studies reported on the detection/quantification of few copies of viral DNA from different high risk/low risk HPV types employing ddPCR. The RT-qPCR or ddPCR testing can provide the most informative prognostic information for oncogenic HPV on patients with head and neck SCC [28,29,30].

The receptor for the virus to enter cells is integrin A6. The receptor involved in virus entry to the host cells is integrin A6 and HPV DNA is found in apoptotic epithelial cells of the oral mucosa of clinically healthy individuals. The HPV DNA was found in apoptotic epithelial cells of the oral mucosa of clinically healthy individuals. They play an important role in the disorders of intercellular connections. They also transmit information related to cell survival. In a SCC immunohistochemical study, immunohistochemically integrin α6β4 has been shown to be involved in early recurrence and tumor remodeling. The expression of α6β4 is lower in squamous cell carcinoma SCC than in the normal epithelium [25,31,32,33,34,35,36]. Transmission occurs between the epithelial surfaces through apoptotic cells containing infectious viruses. So, this leads to the conclusion that the virus is transmitted by salivary route. The fact is this is an indication of the transmission of the virus through saliva. Vertical transmission of the virus from mother to fetus has not been documented (an exception is juvenile respiratory papilloma) [37]. There is a wide variation around 5–12% in the detection of HPVs in the normal oral mucosa/oropharynx, depending on the various techniques (PCR, SB, ISH: in situ hybridization) with a prevalence in male and in certain individuals of HPV 16. We must mention that HPV DNA was found in apoptotic epithelial cells of the oral mucosa of clinically healthy individuals. The prevalence of HPV in the oral cavity of healthy asymptomatic individuals is estimated from 0 to 81% while in children from 0.25 to 48%. It omitted to mention that HPV was detected in leukoplakia lesions in 10%, in verrucous hyperplasia in 13.3 to 27.3% [38].

## 4. The Biomolecular Pathways of Carcinogenesis in HPV Infection

### 4.1. Genomic Organization

Viral DNA is associated with cell histone proteins to form a chromatin-like complex. Regions of attack on histone proteins are the scaffold attachment regions (SARs) or matrix attachment regions (MARs). The HPV genome organized into three unequal regions: E (early), L (late) region, and the non-coding LCR. The E region represents more than 50% of the genome and is responsible for regulating transcription, virus replication, and activating the lytic cycle [39,40]. These regions code respectively early proteins E1, E2, E4, E5, E6, E7 (necessary for viral DNA replication and for the carcinogenesis), two further proteins, E3 and E8 (still unknown function), and for the late proteins L1, L2 belonging to the capsid and having a regulatory function. Thus, open reading frames ORFs that encode the early region (from E1 to E8) are eight and for L1 and L2 they contain the origin of replication and some control elements for transcription and replication and are limited to the single strand. The L region contains 40% of the genome, is responsible for the synthesis of the corresponding structural proteins of the virus and contains the 10–12% of the genome responsible for the expression of the genes of the virus. Finally, the genes that encode the virus proteins are contained in ORFs in the first two regions of the genome [39,41].

### 4.2. HPV Proteins

The E1 is required for virus replication. The E2 binds to E1 and together they activate the replication of viruses. The E4 contributes to facilitate and improve viral replication (controlling the maturation and release of the virus) and is the first expressed in the late stages of infection and is subsequently followed by L1 and L2 proteins (expressed specifically in the granular layer of the epithelium). In fact, E4 protein can be as an important biomarker in diagnosis of the infection [18,42]. The E5 acts in the early stages of the infection. It can activate the epidermal growth factor receptor (EGFR) and cell proliferation. The transmembrane growth factor receptor EGF (EGFR) binds to EGF family growth factors and is activated. The result of this activation is the transmission of signals from the extracellular space to the cytoplasm. These signals are directly related to the ability of epithelial cells to proliferate independently [43]. The ERbB1 gene, responsible for encoding the EGFR threshold, is in the short arm of chromosome 7p12 and has been found to show increased activity in oral cancer [44]. Modified function of ERbB1 in SCC of the head and neck is attributed more to its overexpression than to the presence of mutations [45]. In parietal and cellular level, overexpression of ERbB1/EGFR is associated with a low degree of differentiation, increased proliferation, inhibition of cancer cell apoptosis, and severe neo-angiogenesis. This translates into tumor aggression, invasion into adjacent tissues and induction of distant metastases, respectively [46]. In cases of SCC of the mouth, studies have shown the presence of a mutation in the 7p12 chromosome [35,47]. Thus, in these cases, the infiltration of the lymph nodes as well as the recurrence of the cancer was increased (worse prognosis) while in the absence of mutation, the survival time of the patients was longer. EGFR is further directly related to the activation of various intracellular transmission pathways, the most important of which are MAPK, Akt-mToR, STAT. Finally, it can also interact with other pathways such as E-cadherin/β-catenin and desoglein/γ-catenin. In the early stages of viral infection, E2 inhibits the transcription of the oncogenes E6 and E7, the two oncoproteins that take part in the carcinogenesis process [46,48,49].

### 4.3. Mechanisms of HPV-Driven Transformation

Thus, the oncogenic activity of the virus is due to the ability of the viral proteins E6 and E7 to integrate into host DNA. These two proteins promote tumor growth by inactivating two tumor suppressor proteins retinoblastoma pRb and tumor protein 53 (gene TP53), that regulate the transition from the G1 phase of the cell cycle to S [50,51]. Cell division follows a predetermined phase evolution. It is interrupted at a specific control point, through various mechanisms if there is a disturbance. These two proteins promote tumor growth by inactivating the two tumor suppressor proteins, pRb retinoblastoma and tumor protein 53 (TP53 gene), which regulate the transition from phase G1 of the cell cycle to phase S [50,51]. It is known that in cell division, a predetermined phase evolution follows and can be interrupted in a specific control point, through various mechanisms if there is a disturbance. A typical example of such a self-limiting mechanism is the retinoblastoma protein pRb pathway. In the absence of cell proliferation, non-phosphorylated pRb complexes with E2F regulatory proteins, which stop the cell cycle in the G1 phase. In the absence of cell proliferation, the non-phosphorylated pRb joins, creating complexes with the E2F regulatory proteins, which interrupt the cell cycle in the G1 phase. When tissue is needed for new cells, in order to increase or replenish a loss, neighboring cells usually produce signals through growth factors (TGF-EGF), which cause phosphorylation of Rb and release of E2F, not E2F, activates the genes responsible for DNA synthesis, by activating c-MYC, p21WAF-1. When it is necessary in the tissues to increase or recover cell loss, neighboring cells produce signals for the activation of growth factors (TGF-EGF), causing Rb phosphorylation and E2F release. As a result, E2F activates the genes responsible for DNA synthesis, through the activation of c-MYC and p21WAF-1. It should be noted that phosphorylation involves Cd-kinases 2, 4, 6 which are complexed with cyclin E and D1 and are regulated by a family of inhibitors, Cd-kinases (CDKIs), p21 and INK4A (p16). There is a gradual loss of pRb expression in dysplastic lesions and especially during malignancy [49,50]. In this process, it probably becomes important that in Path 52 are the possible genetic changes, leading to loss of pRb expression and the phosphorylation-dephosphorylation mechanism of pRb regulation. Phosphorylation-dephosphorylation disorder of pRb is an early event. In conclusion, there is the hypothesis that the retinoblastoma protein pathway is probably involved in carcinogenesis by inactivating cell cycle regulators i.e., cyclin D1, CDK6, p21WAF-1, and p16INK4A [50,51,52,53,54]. In conclusion, it is understood that the retinoblastoma protein pathway is thought to be potentially involved in carcinogenesis by inactivating cyclin D1, CDK6, p21WAF-1, p16INK4A, regulators of cell cycle [36,37,38].

**Figure 3:**
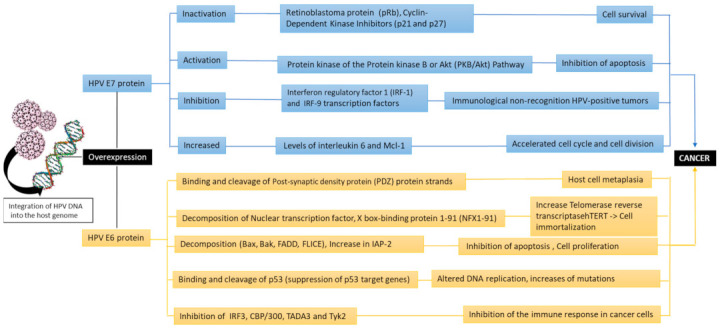
The oncogenic pathways of the HPV due to the activity of the viral oncoproteins E6 and E7 (Bak: Bcl-2 homologous antagonist/killer, FADD: Fas-associated protein with death domain, FLICE: procaspase 8, IAP-2: Interferon regulatory factor 3,CBP/300: CREB binding protein, TADA3: Transcriptional adaptor 3, Tyk2: tyrosine kinase 2).

Overexpressed E6 binds and cleaves the post-synaptic density proteins (PDZs) strands (such as hDlg, hScribble, Multi-PDZ domain protein (MUPP1), protein tyrosine phosphatase non-receptor type 13 (PTPN13), PATJ, and MAG1 (membrane-associated guanylate kinase, WW, and PDZ domain-containing protein 1) that lead to the host cell metaplasia. The E6 inhibits apoptosis, impairs the transcription process, and increases the life cycle of cells. The E6 in turn participates in the cleavage of “wild” type of p53 through a three-dimensional complex consisting of E6, E6AP (E6 associated protein), and TP53 [51,53]. Furthermore, E6 inhibits the action of the tumor protein (p73), a homologous protein of p53. The E6 of HPV type 16 prevents apoptosis by means of a mechanism involved in inhibiting the expression of the apoptosis regulator BAX (bcl-2-like protein 4 Bax) gene in keratinocytes, resulting in the accumulation of DNA. The apoptosis process is also inhibited by binding of E6 to the tumor necrosis factor 1 receptor (TNFR1) [54,55]. E6 is not limited to the above activities, which include the “immortalization” of host cells, but it also has a variety of other effects with similar effects that favor carcinogenesis. It also can bind to the tumor suppressor protein p53, causing it to cleave via a *Ubiquitin ligase* [55]. Instead, E7 binds to the pRb family proteins and interacts with various proteins, regulating cell growth (mainly from the G1 phase to the S cellular phase) and thus dysregulates the cell cycle, increasing the proliferation process with possible cellular malignant transformation [56]. E7 is also associated with a variety of other factors and causes the degradation of pRb. In addition, E7 binds to the active form of pRb, inhibiting its interaction with the transcription factor E2F. Then, in cells characterized by E7 overexpression, a loss of control of the transition from G1 to S phase is observed, resulting in successive continuous cell cycles and uncontrolled proliferation [57]. The P53 levels in normal cells are extremely low, whereas during E7 overexpression, gene TP53 levels rise due to inhibition of its breakdown in normal cells which is regulated by the mediator of DNA damage-binding protein 2 (DDB2). Furthermore, the cyclin dependent kinase inhibitors (p27 and p21) are responsible for promoting continuous cell cycles. In fact, E7 forms complexes with p7b, p107, and p130 proteins (Figure 3) [58,59,60].

Finally, the proteins E6 and E7 responsible for carcinogenesis modulate the epigenetic mechanisms. Epigenetic dysregulation is the change in gene expression that is not caused by variations in the DNA sequence. Epigenetic is represented by three main processes: histone modifications, non-coding RNA, and DNA methylation. Together, these mechanisms can exert their effects by directly regulating DNA transcription, damage/repair and replication, controlling RNA levels and post-transcriptional stability, normalizing protein translation, or causing post-translational changes in proteins [14,61]. Thus, HPV modulates various epigenetic mechanisms including DNA methylation, histone modification, chromatin remodeling, and miRNAs, and can occur on cellular or viral genes (especially when virus is integrated into the host genome) through his oncoproteins E6/E7. Indeed, HPV DNA can be differentially methylated as E6/E7 regulatory mechanism. Hence, the two oncoproteins (E6/E7) induce the expression of DNMT which lead to aberrant DNA methylations by blocking the normal epigenetic processes. Furthermore, E7 binds directly and controls the methyl transferase activity of the enzyme [61,62].

There is increasing evidence that HPV can act in combination with cigarette smoking and alcohol to cause oral cancer [18,63]. The SCC has been found to affect the highest frequency (69.9%) of people using tobacco that is smoked or smoked without smoking [63]. According to these studies, the complete study of hygiene and leukoplakia lesions in the mouth are predisposing factors for the development of oral cancer. However, it has recently been found that HPV-positive oral cancers typically occur in young patients who do not smoke or consume alcohol, as opposed to HPV-negative oral cancers that occur in older smokers and drinkers [64]. The HPV and in particular subtypes 6,11,16, and 18 have been implicated in the lesion onset [65]. The most widely accepted hypothesis emphasizes that HPV infection in combination with chronic tobacco use or alcohol is the most likely cause of SCC [66]. Cigarette smoke releases (due to combustion) a series of chemical products (volatile and non-volatile). From these substances, the main carcinogens are nitrosazines, benzopyrene, diethylbenzanthracene, and free radicals. Benzopyrene is processed by the cytochrome P450 enzyme in mucosal stem keratinocytes to form dihydrodiode epoxides, which can bind to the N2 position of guanine DNA strands and cause aligned mutations [67,68]. On the other hand, nitrosamines (N-nitroso-nicotine and 4 methyl-nitrosazino-pyrido-butanone) produced by nicotine nitration can mutate the DNA of the epithelium with cancer-causing mutations. Thus, there may be possible mutations of the 3 Ras genes (HRas, KRas, NRas), the most common oncogenes, causing a dysregulation of the protein that regulates the pathways for cell differentiation and proliferation. On the other hand, large amounts of alcohol can cause deprivation of the mitochondrial aldehyde dehydrogenase ALDH 2 allele gene and show elevated levels of serum acetaldehyde (produced by the metabolism of ethanol and is converted into acetic acid by ALDH) and salivary, thus increasing the risk of carcinogenesis.

Finally, the oral microbiota eubiosis plays an essential role in maintaining health in the oral cavity and in the general physiology of the human body. Several viruses are present in the oral cavity, such as HPV, Paramyxoviridae (mumps virus), Rhabdoviridae, and others that are mainly related to mouth and upper airways diseases. Most of the virus sequences detected were like those of bacteriophages, which may not be surprising given the density and variety of the bacterial community found in the mouth. To date, the role of a probable interaction between them has not been thoroughly studied with the beneficial role of probiotics in microbiota dysbiosis [69,70,71,72]. 

## 5. Conclusions

From literature data, it appears that (HPV) infection plays an important role in the risk of precancerous lesions of the oral mucosa, and therefore in the dysplastic and malignant transformation of the lesions. Furthermore, the ability of external carcinogens such as smoking, and alcohol may be risk cofactors in the development of carcinogenesis. Indeed, the potential role of HPV in oncogenic processes in the oral cavity remains an element of update regarding prevention and treatment. HPV is then associated with malignant evolutions and, therefore, with different types of the oropharynx. Thus, carcinogenic HPVs such as 18, 56, 58, and others have been implicated in the pathogenesis from studies conducted in the onset of the cancerous lesion. Therefore, also predisposing factors for the development of cancer can be hygiene and dysbiosis of the oral microbiota. The most widely accepted hypothesis by scientists points to occasional HPV infection in combination e.g., with chronic tobacco or alcohol use as the most likely cause of oral cancer pathogenicity along with genetic predisposition.
